# Apoptosis resistance in HIV-1 persistently-infected cells is independent of active viral replication and involves modulation of the apoptotic mitochondrial pathway

**DOI:** 10.1186/1742-4690-5-19

**Published:** 2008-02-08

**Authors:** Pablo N Fernández Larrosa, Diego O Croci, Diego A Riva, Mariel Bibini, Renata Luzzi, Mónica Saracco, Susana E Mersich, Gabriel A Rabinovich, Liliana Martínez Peralta

**Affiliations:** 1 National Reference Center for AIDS, Department of Microbiology, School of Medicine, University of Buenos Aires, Buenos Aires, Argentina; 2 Laboratory of Immunopathology, Institute of Biology and Experimental Medicine (IBYME), CONICET, Buenos Aires, Argentina; 3 Laboratory of Virology, Department of Biochemistry, School of Exact and Natural Sciences, University of Buenos Aires, Buenos Aires, Argentina

## Abstract

**Background:**

HIV triggers the decline of CD4^+ ^T cells and leads to progressive dysfunction of cell-mediated immunity. Although an increased susceptibility to cell death occurs during the acute phase of HIV infection, persistently-infected macrophages and quiescent T-cells seem to be resistant to cell death, representing a potential reservoir for virus production.

**Results:**

Lymphoid (H9/HTLVIII_B _and J1.1) and pro-monocytic (U1) HIV-1 persistently-infected cell lines were treated with hydrogen peroxide (H_2_O_2_) and staurosporine (STS) for 24 h, and susceptibility to apoptosis was evaluated and compared with uninfected counterparts (H9, Jurkat and U937 respectively). When exposed to different pro-apoptotic stimuli, all persistently-infected cell lines showed a dramatic reduction in the frequency of apoptotic cells in comparison with uninfected cells. This effect was independent of the magnitude of viral replication, since the induction of viral production in lymphoid or pro-monocytic cells by exposure to TNF-α or PMA did not significantly change their susceptibility to H_2_O_2_- or STS-induced cell death. A mechanistic analysis revealed significant diferences in mitochondrial membrane potential (MMP) and caspase-3 activation between uninfected and persistently-infected cells. In addition, Western blot assays showed a dramatic reduction of the levels of pro-apototic Bax in mitochondria of persistently-infected cells treated with H_2_O_2 _or STS, but not in uninfected cells.

**Conclusion:**

This study represents the first evidence showing that resistance to apoptosis in persistently-infected lymphoid and monocytic cells is independent of active viral production and involves modulation of the mitochondrial pathway. Understanding this effect is critical to specifically target the persistence of viral reservoirs, and provide insights for future therapeutic strategies in order to promote complete viral eradication.

## Background

Apoptosis represents a type of programmed cell death (PCD) occurring in various physiological and pathologycal processes. The ability of a cell to undergo or resist apoptosis in response to viral infection is crucial in determining the clinical outcome of the disease and its therapeutic oportunities [1,2]. Human imunodeficiency virus (HIV) is the causative agent of acquired immunodeficiency syndrome (AIDS), which triggers the decline of CD4^+ ^T cells and leads to immune system dysfunction [3,4]. During HIV-1 infection, most apoptotic events predominantly occur in uninfected bystander T cells through indirect mechanisms, such as the Fas/Fas ligand and CXCR4/CD4-mediated pathways [5,6]. However, acutely-infected CD4^+ ^T cells are susceptible to dying by apoptosis, by direct cell cytotoxicity induced by HIV replication, superantigen-induced cell death, immune-mediated killing involving cytotoxic T-lymphocytes (CTL), antibody-dependent cell cytotoxicity (ADCC) or syncytia formation [7].

However, in some circumstances, HIV-infected cells do not seem to undergo apoptosis following infection and these cells have been proposed to play an important role as viral reservoirs. Persistently-infected pro-monocytic, but not lymphoid cell lines have been shown to be less sensitive to several apoptotic stimuli when compared with their uninfected counterparts [8]. Besides, chronically-infected macrophages and quiescent T cells seem to be resistant to cell death, thus representing a potential reservoir for viral production which might favor viral spread to other susceptible target cells [5,9,10]. The survival of productively-infected CD4^+ ^lymphocytes or T cell lines was found to be influenced by viral proteins when exposed to apoptotic stimuli [11-13].

However, in spite of the relevance of these reservoir cells in the control of viral persistence, the mechanisms responsible of apoptosis resistance of persistently-infected cells are not well understood. In particular, it is still unclear whether resistance of infected cells to apoptotic stimuli involves modulation of active viral replication. In the present study, persistently-infected pro-monocytic and T-cell lines and their uninfected counterparts were treated with H_2_O_2 _or STS. These apoptotic stimuli were selected according to their ability to induce apoptosis via reactive oxygen species (ROS) [14] and protein kinase C (PKC) inhibition [15], which lead to an increase of oxidative stress. These stimuli generate a cell state which resembles the typical phenotype of cells undergoing active viral replication and antiretroviral treatment [16,17]. When treated, all persistently-infected cells showed significantly lower frequency of apoptotic cells when compared with those uninfected, independently of the magnitude of viral production. In addition, resistance to apoptosis induced by HIV involved modulation of mitochondrial Bax expression in persistently-infected cells.

## Results

### HIV-1 persistently-infected cell lines are resistant to apoptosis induced by H_2_O_2 _and STS

**Figure 1:**
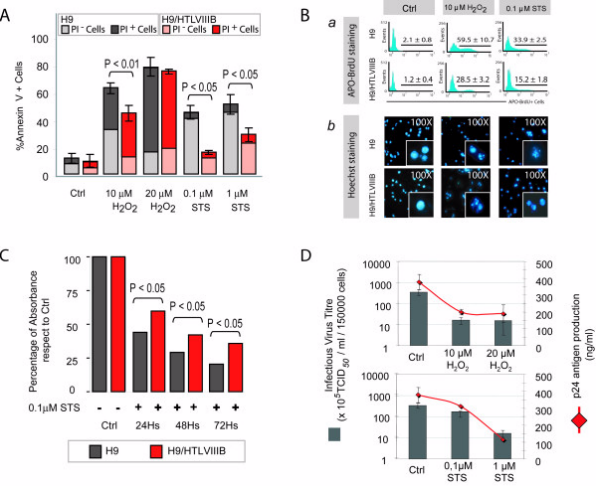
**Apoptosis resistance in HIV persistently-infected H9/HTLVIII_B_cells in comparison with non-infected H9 cells**. **A**) H9 and H9/HTLVIII_B _cells were treated with different concentrations of H_2_O_2 _or STS or complete medium as control. After 24 h, cells were harvested and annexin-V/PI staining was performed. The percentages of annexin-V^+^, PI^- ^or PI^+ ^cells are shown. **B**) (a) Analysis by APO-BrdU labeling by flow cytometry. The corresponding histograms and the percentages of APO-BrdU^+ ^cells are shown; (b) Analysis of apoptosis with Hoechst 33324 by fluorescence microscopy. Micrographs (100×) of predominant Hoechst stained nuclei are depicted. **C**) H9 and H9/HTLVIII_B _cells were treated with 0.1 μM STS or complete medium as control, and cells were harvested 24, 48 and 72 h post-treatment. Cell viabillity was analyzed by the MTT assay. Absorbances from treated samples were normalized to 100% of untreated controls. **D**) Cells treated with H_2_O_2 _or STS or complete medium for 24 h were pelleted and the supernatants were used to quantify infective viral (grey bar) and p24 antigen (red line) production.

Uninfected H9 and persistently-infected H9/HTLVIII_B _cells were cultured with RPMI 1640 complete medium in a humidified atmosphere (5% CO_2 _in air) at 37°C and incubated with different concentrations of H_2_O_2 _and STS. Simultaneously, cells were incubated with medium alone and used as controls. Cells were collected 24 h post-treatment, and apoptosis was evaluated by annexin-V/propidium iodide (PI) and APO-BrdU staining. Treatment with 10 μM H_2_O_2 _induced 35% of annexin-V^+^/PI^- ^H9 cells, and 15% of annexin-V^+^/PI^- ^infected H9/HTLVIII_B _cells (*P *< 0.01), whereas 20 μM H_2_O_2 _induced massive death in both cell lines, characterized by predominant necrosis (60–65%) and lower levels of apoptosis (18–20%) (Figure 1A). On the other hand, treatment with 0.1 μM STS induced 43% of apoptotic H9 cells, whereas the frequency of annexin-V^+^/PI^- ^cells was only 15% in the infected H9/HTLVIII_B _cells (*P *< 0.01). These differences were also observed when concentrations of 1 μM STS were used to promote cell death (Figure 1A). Furthermore, differences in the levels of apoptosis between infected and uninfected cells were confirmed when cells were exposed to 10 μM H_2_O_2 _or 0.1 μM STS and stained with APO-BrdU and Hoechst 33324 (Figure 1B).

Cell viability was assessed by the MTT assay and absorbances were measured at 540 nm, normalized against controls (Ctrl) and expressed as percentages. The percentage of viable cells was found to be 34% when H9 cells were treated with 10 μM H_2_O_2_, but reached percentages of 50% in H9/HTLVIII_B _cells. Furthermore, treatment with 0.1 μM STS showed a decrease of MTT levels up to 48% and 64% in H9 and H9/HTLVIII_B _cells respectively (data not shown). MTT was also assessed in both cell lines treated with 0.1 μM STS for 24, 48 and 72 h, indicating differences in cell viability of both cell lines that were still significant until day 3 post-treatment (Figure 1C).

In order to investigate the association between apoptosis and viral production in H9/HTLVIII_B _cells, p24 antigen, viral load and production of infectious viral particles were quantified. The magnitude of decrease of p24 antigen production observed was 80% (119 ng/ml), 75% (189 ng/ml), 78% (312 ng/ml) and 23% (114 ng/ml), when H9/HTLBIII_B _cells were treated with 10 μM H_2_O_2_, 20 μM H_2_O_2_, 0.1 μM STS and 1 μM STS respectively and compared with controls (H_2_O_2_: 254 ng/ml; STS: 398 ng/ml) (Figure 1D). Viral load values were similar to p24 antigen levels (data not shown). Infectious virus titres were also in agreement with p24 levels when cells were treated with apoptosis inducers (Figure 1D).

**Figure 2:**
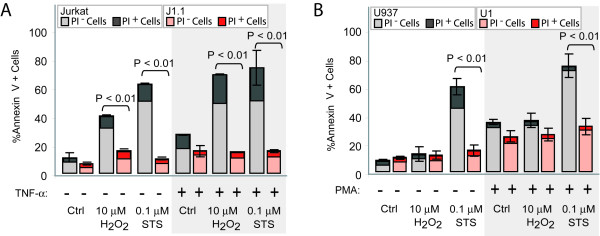
**Apoptosis resistance is independent of the magnitude of viral replication**. **A**) Jurkat and J1.1 cells were incubated in the presence or absence of 1000 U/ml TNF-α for 48 h, and then treated with 10 μM H_2_O_2 _or 0.1 μM STS. The percentages of annexin-V^+^, PI^- ^or PI^+ ^cells are shown. **B**) U937 and U1 cells were incubated in the presence or absence of 100 ng/ml PMA for 24 h, and then exposed to 10 μM H_2_O_2 _or 0.1 μM STS. The percentages of annexin-V^+^, PI^- ^or PI^+ ^cells are shown.

To examine whether apoptosis resistance in persistently-infected cells was dependent of the nature of the cell lines tested, experiments were carried out using the lymphoid Jurkat T-cell line and its infected counterpart (J1.1), or in the pro-monocytic U937 cell line and its infected U1 counterpart. The percentage of annexin-V^+^/PI^- ^cells was 30% and 47% when Jurkat T cells were treated with 10 μM H_2_O_2 _and 0.1 μM STS, respectively, and only 8% and 6% for J1.1 cells exposed to these apoptotic stimuli (Figure 2A). Regarding the pro-monocytic U937 cell line and its infected counterpart U1, an important increase was observed in the percentage of annexin-V^+^/PI^- ^cells (45%) in U937 cells treated with 0.1 μM STS, but only 8.2% in U1 infected cells. Remarkably, no significant differences were observed in the frequency of apoptosis when cells were treated with 10 μM H_2_O_2 _(Figure 2B). However, a higher concentration of H_2_O_2 _(50 μM) was capable of inducing 34% of annexin-V^+ ^U937 cells, and only 16.5% of dead cells in the infected U1 cell lines (data not shown). This result could be explained by the fact that pro-monocytic cells are substantially less susceptible to experience damage by ROS [18]. Thus, lymphoid and pro-monocytic HIV-1 persistently-infected cell lines are less susceptible to apoptosis induced by H_2_O_2 _or STS treatment compared with their uninfected counterparts.

### Apoptosis resistance of HIV-infected cell lines is independent of the magnitude of viral production

Unlike H9/HTLVIII_B_, J1.1 and U1 cell lines are non-productive cells unless treated with a viral activator. To investigate the differential sensitivity to apoptosis of infected cells under conditions of active viral replication, Jurkat and J1.1 cells were treated with 1000 U/ml tumor necrosis factor-α (TNF-α) for 48 h and U937 and U1 cells were treated with 100 ng/ml phorbol-12-myristate-13-acetate (PMA) for 24 h. Active viral production was confirmed by determining the p24 antigen at different days post-treatment. TNF-α treatment induced 100-fold viral reactivation at 48 h with respect to untreated cells, while U1 cells showed 50-fold and 200-fold increase of viral production at 24 h and 48 h, respectively, when cultured with PMA (Table I). Under these conditions, the percentage of annexin-V^+^/PI^- ^cells was 48% and 52% when Jurkat cells were treated with 10 μM H_2_O_2 _and 0.1 μM STS, respectively, and only 12% for J1.1 cells exposed to both apoptotic stimuli (Figure 2A).

**Table 1:** P24 production in HIV-1 persistently-infected cell lines exposed to TNF-α or PMA

**Cell line and treatment**	**0 h**	**24 h**	**48 h**
**J1.1 Cells**	14.07 ± 0.01	14.14 ± 0.05	18.26 ± 0.80
**J1.1 Cells + TNF α**	14.08 ± 0.01	12.86 ± 0.90	152.04 ± 1.50
**U1 Cells**	1.06 ± 0.03	1.60 ± 0.03	4.18 ± 1.11
**U1 Cells + PMA**	1.05 ± 0.03	51.96 ± 9.20	191.76 ± 0.48

HIV-1 persistently-infected lymphoid J1.1 and monocytic U1 cells were treated with TNF-α and PMA respectively in order to stimulate viral replication. P24 antigen was determined at 0, 24 and 48 h in cell supernatants by ELISA (HIVAG-1 Monoclonal, Abbot Laboratories). Values correspond to p24 antigen per 200,000 cells, expressed in J1.1 as pg/ml and in U1 Cells as ng/ml.

Regarding the pro-monocytic cell lines, when these cells were pre-incubated with PMA, the frequency of early apoptotic cells was significantly increased in both cell lines treated with STS: 72% in U937 and 30% in U1 cells (Figure 2B). Control cells showed also higher levels of apoptosis when pre-incubated with PMA. It should be emphasized that PMA, independently of its ability to stimulate viral replication, can also induce cell differentiation, an effect which can influence the susceptibility to apoptosis [19]. These data suggest that apoptosis resistance in persistently-infected cell lines is independent of the magnitude of viral replication.

### Apoptosis resistance of HIV persistenly-infected cell lines involves modulation of the mitochondrial pathway

**Figure 3:**
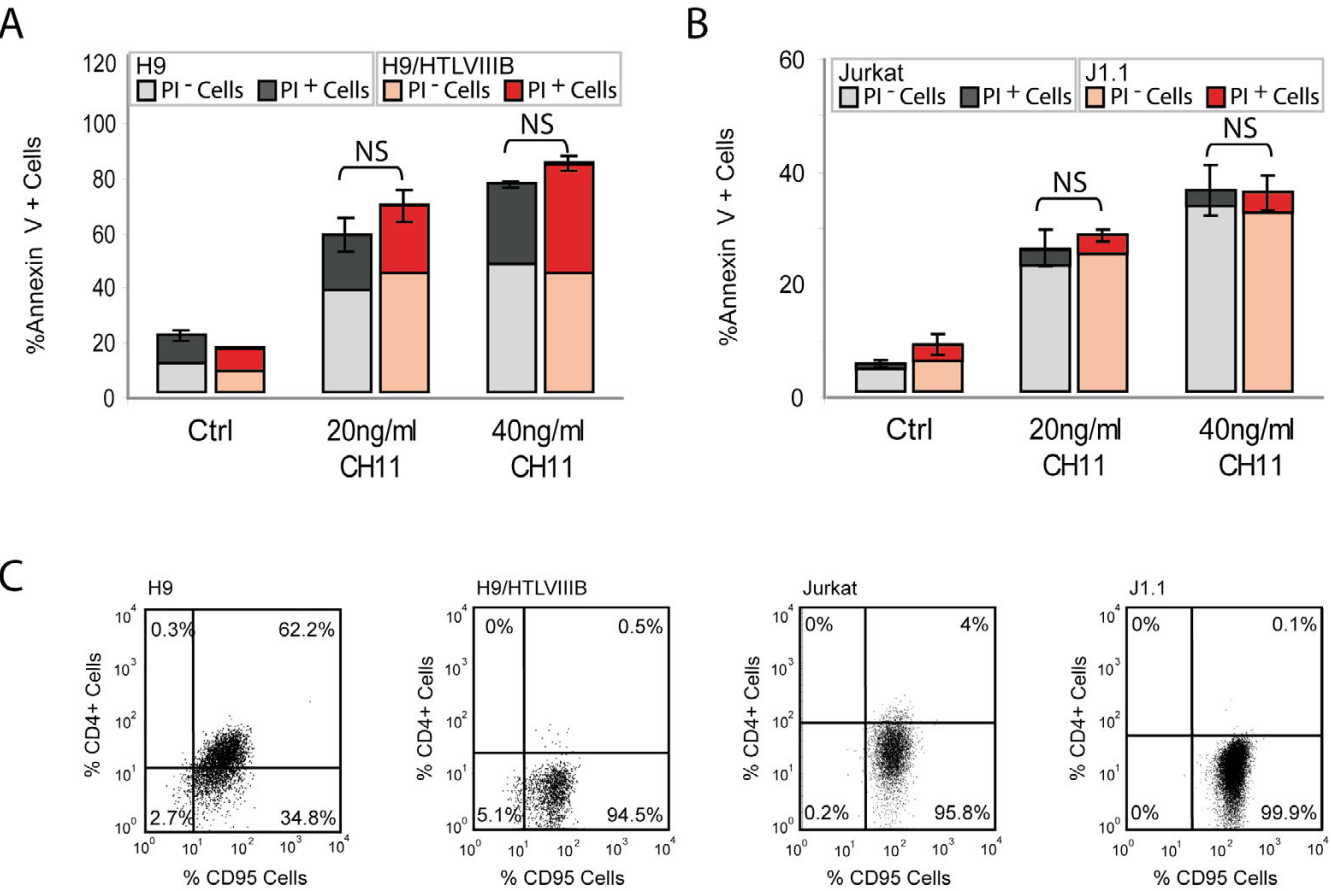
**Fas-mediated apoptosis in uninfected and HIV persistently-infected cells**. H9 and H9/HTLVIII_B _(**A**) and Jurkat and J1.1 (**B**) cells were incubated with 20 g/ml or 40 ng/ml of CH11, an anti-Fas activating antibody. After 24 h, cells were washed and stained with annexin-V/PI. The percentages of annexin-V^+^, PI^- ^or PI^+ ^cells are shown. **C**) Fas/CD95 and CD4 cell surface expression was analyzed by flow cytometry on H9, H9/HTLVIII_B_, Jurkat and J1.1 cells.

In order to dissect the mechanisms involved in this protective effect, uninfected or persistently-infected cell lines treated or not with apoptotic stimuli were used to analyze different apoptotic pathways. First, the anti-Fas activating antibody CH11 was used to induce apoptosis by the extrinsic pathway in H9 and H9/HTLVIII_B _cells, and Jurkat and J1.1 cells. No significant differences were observed between uninfected and persistently-infected cells (Figure 3A–B). As Fas/CD95 expression was found to be modulated by HIV-1 [5], we examined cell surface expression of Fas antigen in order to check whether our results could be due to differential expression of this receptor. Flow cytometry analysis revealed no significant differences of Fas expression among all cell lines tested (Figure 3C). Thus, HIV-1 persistent infection does not seem to modulate the susceptibility to apoptosis by controlling the extrinsic pathway.

**Figure 4:**
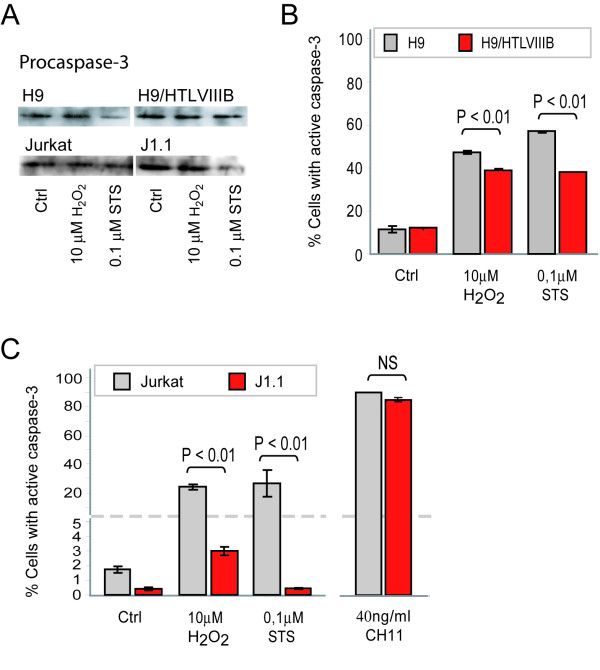
**Caspase-3 activation by H_2_O_2 _and STS treatment in uninfected and HIV persistently-infected lymphoid cell lines**. **A**) H9, H9/HTLVIII_B_, Jurkat and J1.1 cells were exposed to H_2_O_2 _and STS. After 24 h, cells were washed and lysed with RIPA buffer. Equal amounts of protein (30 μg/sample) were separated on a 10% SDS-PAGE and blotted onto nitrocellulose membranes. Blots were probed with anti-procaspase-3 for 1 h and revealed with a peroxidase-conjugated anti-IgG antibody and ECL (enhanced chemoluminiscence) Equal loading was checked by analyzing β-actin expression (data not shown). **B**) H9 and H9/HTLVIII_B _cells were exposed to 10μM H_2_O_2_, 0.1 μM STS or complete medium for 24 h and collected to evaluate active caspase-3 by PE-conjugated monoclonal anti-active caspase-3 antibody by flow cytometry. **C**) Jurkat and J1.1 cells were exposed to 10 μM H_2_O_2_, 0.1 μM STS, 40 ng/ml CH11 or complete medium for 24 h and collected to evaluate active caspase-3 by PE-conjugated monoclonal anti-active caspase-3 antibody by flow cytometry as described in *Materials and Methods*

To gain insight into the mechanistic basis of this effect, we next analyzed events associated with the execution of apoptosis. When procaspase-3 expression was evaluated by Western blot analysis in H9 and H9/HTLVIII_B_, or Jurkat and J1.1 cells, no significant differences were observed in untreated controls. However, when treated with the pro-apoptotic agents, a decrease of procaspase-3 was observed in all the cases (Figure 4A). When cells were analyzed by flow cytometry, H9 cells were 57% and 47% positive for active caspase-3 when treated with H_2_O_2 _and STS respectively, while H9/HTLVIII_B _raised percentages of 39% and 38% respectively (Figure 4B). Besides, Jurkat cells showed even higher differences in caspase-3 activation than J1.1 when treated with H_2_O_2 _(Jurkat: 24%; J1.1: 3%) and STS (Jurkat: 25.13% ;J1.1: 0.43%) (Figure 4C). Furthermore, when treated with 40 ng/ml CH11 anti-Fas antibody, the number of cells with active caspase-3 was similar in both uninfected and persistently-infected cells (Figure 4C). Taken together, these results suggest that differences in the susceptibility to apoptosis between infected and uninfected cells can not be explained by defective caspase-3 activation and that apoptosis modulation may be localized upstream of caspase-3.

**Figure 5:**
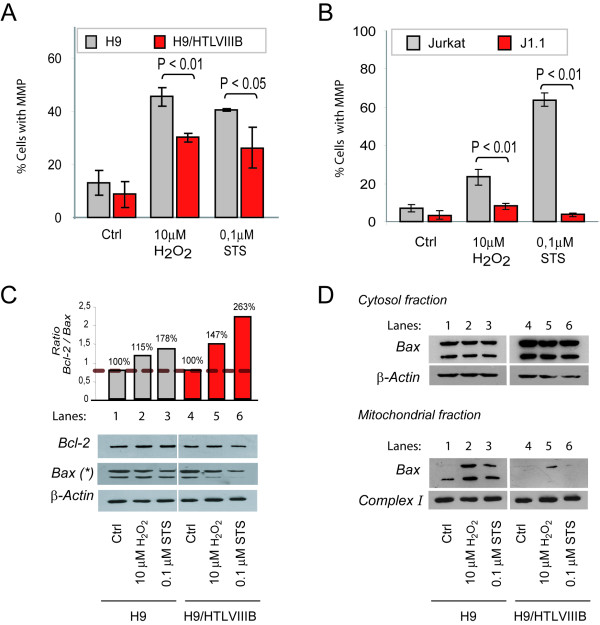
**MMP induction and *Bcl-2 *and *Bax *expression in uninfected or HIV persistently-infected cell lines**. H9 and H9/HTLVIII_B _(**A**) and Jurkat and J1.1 (**B**) cells were exposed to 10 μM H_2_O_2_, 0.1 μM STS or complete medium for 24 h and harvested to evaluate mitochondrial membrane potential (ΔΨm) by JC-1 staining by flow cytometry. **C**) H9 and H9/HTLVIII_B _cells were exposed to H_2_O_2 _and STS. After 24 h, cells were washed and lysed with RIPA buffer. Equal amounts of protein (30 μg/sample) were separated by 10% SDS-PAGE and blotted onto nitrocellulose membranes. The blots were probed with anti-Bcl-2 and anti-Bax antibody, revealed using a peroxidase-conjugated anti-IgG and developed using a chemiluminiscence Western blotting detection reagent. Equal loading was checked by analyzing β-actin expression. Films were analyzed with Scion image analysis software (Scion, Frederick, MD) and the Bcl-2/Bax ratio was depicted. **D**) H9 and H9/HTLVIII_B _cells were exposed to H_2_O_2 _and STS and after 24 h lysates from cytosolic and mitochondrial fractions were prepared by differential centrifugation. Equal amounts of protein (30 μg/sample) were separated by 10% SDS-PAGE and blotted onto nitrocellulose membranes. Blots were then probed with an anti-Bax polyclonal antibody, incubated with a peroxidase-conjugated anti-rabbit secondary antibody and developed using ECL detection reagent. Equal loading was checked by analyzing β-actin (cytosol fraction) and Complex I (mitochondrial fraction) expression.

To further understand this effect we analyzed events associated with the mitochondrial apoptotic pathway. For this purpose, the mitochondrial membrane potential (MMP) was studied in cells treated with H_2_O_2 _or STS by JC-1 (Mitoscreen, BD) staining and flow cytometry. When cells were treated with 10 μM H_2_O_2 _or 0.1 μM STS for 24 h, H9 and Jurkat cells showed higher MMP (H9: 45% with H_2_O_2 _and 40% with STS; Jurkat: 23% with H_2_O_2 _and 64% with STS) compared with H9/HTLVIII_B _and J1.1 cells respectively (H9/HTLVIII_B_: 30% with H_2_O_2 _and 26% with STS; J1.1: 8% with H_2_O_2 _and 3.6% with STS) (Figure 5A–B).

Finally, Bcl-2 and Bax expression of different uninfected and persistently-infected cell lines was analyzed by Western blot of total cell lysates. Densitometric analysis revealed no significant differences in Bcl-2 (25 kDa) expression levels between H9 and H9/HTLVIII_B _cells, treated or not with H_2_O_2 _or STS. However, dimeric Bax (42 kDa) was decreased by ~40% (H_2_O_2_) and ~70% (STS) in H9/HTLVIII_B _cells treated with pro-apoptotic stimuli in comparison with controls or uninfected H9 cells, which reached values of only 20% (H_2_O_2_) or 40% (STS). The overall effect could be observed by analyzing the Bcl-2/Bax ratio, which estimates the anti-apoptotic/pro-apoptotic balance. When treated with pro-apoptotic stimuli, H9/HTLVIII_B _cells (lanes 5 and 6) showed a higher Bcl-2/Bax ratio compared to H9 cells (lanes 2 and 3) (Figure 5C). In order to confirm this observation, Bax dimerization and insertion in mitochondria was analyzed by Western blot from cytosolic and mitochondrial fractions. While the levels of Bax expression remained unaltered in the cytosolic fraction of different uninfected or infected cell lines, the levels of Bax increased substantially in the mitochondrial fraction of uninfected cells treated with apoptotic agents. However, no significant differences were observed in persistently-infected cells when compared to controls (Figure 5D).

These results suggest that apoptosis resistance observed in persistently-infected cells involves modulation of the mitochondrial pathway.

## Conclusions and Discussion

During the clinical course of HIV-1 infection, the depletion of the CD4^+ ^T cell compartment is mainly explained by apoptosis of uninfected cells due to indirect mechanisms including Fas/FasL interaction, syncytia formation and direct citotoxicity of soluble viral proteins such as gp120, Tat or Nef [5,7]. However, HIV-1 may survive in a latent status, mainly in macrophages, resting CD4^+ ^quiescent T cells and CD44^high ^memory T cells [10,20-22]. These cells appear to be less sensitive to death induced by a variety of apoptotic stimuli such as chronic stress [18], or the Fas/FasL (CD95L) system [23] independently of viral cofactors. Therefore, when the chronic infection is established in macrophages or in memory T cells, the virus may survive longer in these cells due to a variety of cellular and viral factors [24,25].

Our data suggest that persistently-infected pro-monocytic and lymphocytic cells are less susceptible to undergo apoptosis when exposed to different apoptotic stimuli such as H_2_O_2 _and STS, compared with uninfected cells. This protection from apoptosis is consistent with the fact that HIV-1 persistently-infected macrophages, quiescent T cell and pro-monocytic cell lines were described to survive longer [8,10,20-22]. Our study provides the first evidence showing that apoptosis resistance in persistently-infected cell lines is independent of the magnitude of viral replication. In spite of the fact that H9/HTLVIII_B _cells produced virus actively, while viral production in J1.1 or U1 was inducible, all cell lines showed similar tendences in their resistance to apoptosis when compared with their uninfected counterparts.

Viral proteins are known to modulate cell surface levels of Fas and its ability to transduce death signals upon binding its specific ligand [5]. However, similar expression of Fas antigen was found on the surface of the cells studied, whether infected or not. In addition, engagement of Fas by the stimulating CH11 antibody resulted in similar levels of apoptosis in the cell lines studied, suggesting that HIV-1 infection does not modulate the extrinsic pathway of cell death.

In addition, when the MMP was analyzed in cells treated with H_2_O_2_and STS, substantial differences in the induction of apoptosis were observed between uninfected and persistently-infected cells. This result might be explained by the ability of H_2_O_2 _and STS to induce oxidative stress, thus priming cells to undergo apoptosis via the mitochondrial pathway. These results are also consistent with the levels of caspase-3 activation, indicating that once the mitochondrial pore is induced, apoptosis events proceed normally. Thus, modulation of apoptosis might occur before or during pore induction. In order to analyze the possible mechanisms involved in this effect, expression of Bcl-2 and Bax was analyzed in the cytosolic and mitochondrial compartments. Bcl-2 expression did not show any significant difference between both cell lines, whether they were treated or not with pro-apoptotic stimuli. However, expression of Bax was dramatically reduced in mitochondria of persistently-infected cells when apoptosis was induced by exposure to H_2_O_2 _or STS.

It is now widely accepted that persistent HIV-1 infection represents a new homeostatic state of the cell, which is likely promoted by the combination of both cellular and viral factors. Several viral proteins have been recognized by their ability to induce apoptosis in infected or uninfected cells, but some viral proteins can also protect against cell death [5]. Decreased caspase-3 activation [26] and p53 expression [27] were described as possible mechanisms implicated in apoptosis resistance in HIV-1-persistently infected cells. This study provides novel evidence showing that resistance to apoptosis in persistently-infected cells involves direct modulation of the mitochondrial pathway by regulating Bax pore induction. Further experiments are needed in order to clarify the mechanism by which the virus decreases MMP and controls the execution of apoptosis. Viral regulation of autophagy of damaged mitochondrias or Bax proteolysis might be potential explanatory mechanisms for our observations.

The survival of viral reservoirs is a great challenge to tackle regarding HIV eradication. Understanding the mechanistic bases of the resistance to apoptosis is essential to specifically target the persistence of viral reservoirs and might contribute to provide insights for future therapeutic strategies in order to promote complete viral eradication.

## Materials and Methods

### Cell lines

The following uninfected cell lines of human origin were used: lymphocytic H9, Jurkat and promonocytic U937 cell lines; and their respective HIV-1 persistently-infected cell lines: H9/HTLVIII_B_, J1.1 and U1. All cell lines were provided by the NIH AIDS Research and References Reagent Program, except for U937. Cell lines were cultured with RPMI 1640 medium supplemented with 2 mM L-glutamine, 100 μg/ml streptomycin and 10% fetal calf serum at 37°C in a humidified atmosphere (5% CO_2 _in air).

### Antibodies and reagents

Annexin-V apoptosis kit, APO-BrdU apoptosis kit, active caspase-3 antibody kit, JC-1 Mitoscreen, TNF-α, PE-conjugated anti-CD95 and PerCP-conjugated anti-CD4 antibodies were from BD Biosciences,(CA, USA). Anti-Bcl-2 (DC21), anti-Bax (D21), anti-procaspase-3 (L-18), anti-β-actin (I-19) polyclonal antibodies and peroxidase-conjugated anti-rabbit and anti-goat antibodies were from Santa Cruz Biotechnology, (CA, USA). Anti-complex I antibody was a generous gift from Dr. J. Poderoso (Hospital de Clínicas, University of Buenos Aires). Anti-Fas activating antibody (CH11) was from Upstate (New York, USA). Other reagents including Hoechst, MTT, PMA, staurosporine (STS), Kodak BioMax films were from Sigma (St. Louis, MO, USA). Hydrogen peroxide (H_2_O_2_) and isopropanol were from Merck (New Jersey, USA). RPMI 1640 medium, fetal calf serum, L-glutamine and streptomycin were from Gibco (New York, USA). Micro-BCA protein assay kit was from Pierce (Rockford, USA). Chemiluminiscence Western blotting detection reagent and nitrocelulose membranes were from Amersham Biosciences, UK.

### Induction of HIV-1 production

In order to induce viral production, J1.1 cells were incubated for 48 h with 1000 U/ml TNF-α [28] and U1 cells were exposed to 100 ng/ml PMA for 24 h [29]. Cells were washed twice with PBS and fresh medium was added to carry out experiments. Viral production was confirmed by p24 antigen determination.

### Determination of viral production

Cells were pelleted and supernatants were used to quantify p24 antigen with a commercial ELISA kit (HIVAG-1 monoclonal, Abbot Laboratorios, Illinois, USA), and viral load using a commercial assay (Quantiplex XTm HIV RNA 3.0 Assay bDNA, Chiron Corp, CA, USA). Infective virus titration was performed by limiting dilution and syncytia formation in MT-2 cells, and calculated by the Reed & Müench method.

### Induction of apoptosis

Cells were collected, washed with PBS, resuspended with complete medium, and divided in a 24-well culture plate with a final cell concentration of 150,000 cells/ml. For apoptosis induction, H_2_O_2_, STS and the CH11 Fas activating antibody were used. Optimal concentrations for experiments were standarized by testing different concentrations, which ranged from 5 to 1000 μM (H_2_O_2_), from 0.01 to 10 μM (STS) and from 20 to 40 ng/ml (CH11). Treated cells were always compared with untreated controls (Ctrl). In most experiments, cells were incubated with the apoptosis inducers for 24 h

### MTT assay

Cell viability was determined by the MTT (3-[4,4-dimethylthiazol-2-yl]-2,5-diphenyltetrazolium bromide) assay [30]. After 24 h of exposure to pro-apoptotic stimuli, medium was removed and cells were plated at 5 × 10^4 ^cells/well in 96-well plates and incubated with 0.5 mg/ml MTT in RPMI-1640 without Red Phenol for 1 h at 37°C in a CO_2 _incubator. Cells were pelleted and formazan crystals were solubilized with 0.04 M HCl in isopropanol. Finally, the absorbance measured at 640 nm was subtracted from the absorbance at 540 nm. Each assay was performed in triplicate. Absorbances corresponding to treated samples were normalized to 100% of untreated controls and expressed as percentages. In this assay, the number of surviving cells was directly correlated with the amount of formazan obtained.

### Asssesment of apoptosis

Cells were incubated in the presence or absence of proapoptotic stimuli for 24 h, washed twice with PBS and the frequency of apoptotic cells was analyzed by the following methods:

#### Annexin-V/PI staining

To determine the percentage of early apoptotic cells, phosphatidylserine (PS) cell translocation and plasma membrane permeability were evaluated by dual staining with FITC-conjugated annexin-V and propidium iodide (PI) using the Annexin-V/PI apoptosis detection kit (BD Biosciences) and analyzed by flow cytometry using a FACSCanto (BD Biosciences). Annexin-V^+^/PI^- ^cells representing early apoptotic cells, and annexin-V^+^/PI^+ ^mostly representing necrotic cells were determined.

#### APO-BrdU staining

Late apoptotic cells were determined with the APO-BrdU kit by incorporation of bromodeoxyuridine triphosphate (Br-dUTP) to 3'-hydroxyl sites in cell DNA, and analyzed by flow cytometry in a FACSCanto (BD Bioscience).

#### Hoechst 33324 staining

Apoptotic cells were determined by Hoechst staining and visualized in a fluorescence microscope (Axiophot West Germany).

### Cytofluorimetric analysis of MMP

After treatments with H_2_O_2 _and STS for 24 hours, cells were collected and resuspended in PBS, and then stained with JC-1 (5,5',6,6'-tetrachloro-1,1',3,3'-tetraethylbenzimidazolcarbocyanine iodide) (JC-1 Mitoscreen, BD) for 15 min at 37°C in a CO_2 _incubator. Cells were pelleted, washed twice with buffer supplemented by the kit as indicated by the manufacter and analyzed on a flow cytometer (FACSCanto, BD Biosciences).

### Cytofluorimetric analysis of caspase-3 activation

Treated and control cells were pelleted and washed twice with PBS and the percentage of cells with active

caspase-3 was assessed using the PE-conjugated monoclonal active caspase-3 antibody kit (BD Pharmigen) and analyzed on a FACSCanto flow cytometer (BD Biosciences).

### Flow cytometry analysis

In all cases where flow cytometry was required, 20,000 events were acquired in a FACSCanto flow cytometer (BD Biosciences) and different parameters were analyzed using the WinMDI 2.8 software.

### Isolation and purification of mitochondria

Cells (1 × 10^7 ^cells) incubated in the presence or absence of pro-apoptotic stimuli were washed and homogenized in MSHE (0.225 M mannitol, 0.07 M sucrose, 1 mM EGTA, and 25 mM HEPES/KOH; 1/10 w/v; pH 7.4) and centrifuged at 5,500 × g for 10 min at 4°C. The resultant supernatant was centrifuged at 15,000 × g for 20 min at 4°C and the pellet was resuspended in 30 μl of MSHE (mitochondrial fraction) [31]. To remove broken mitochondria, contaminating organelles, and debris from the cytosol fractions, the supernatants were further centrifuged at 21,000 × g for 30 min at 4°C. Protein concentration from cytosolic and mitochondrial fractions was determined by the Micro-BCA protein assay kit (Pierce, Rockford, USA).

### Western blot analysis

After exposure to pro-apoptotic stimuli, cells were lysed in RIPA buffer containing 20 mM Tris-HCl, 150 mM NaCl, 1% Triton X-100, 1% sodium deoxycholate, 2 mM EDTA, 0.1% SDS and protease inhibitor cocktail. Protein concentrations from total, cytosolic or mitochondrial lysates were quantified using the Micro-BCA protein assay kit as described above. Equal amounts of protein (30 μg/sample) were separated in a 10% SDS-PAGE and blotted onto nitrocellulose membranes. Blots were then probed with anti-pro-caspase-3, anti-Bcl-2 or anti-Bax rabbit polyclonal antibodies as described [32], and incubation with peroxidase-conjugated anti-IgG was performed in a blocking buffer for 1 h. Blots were then developed using a chemiluminiscence Western blotting detection reagent and exposed to X-ray films. Films were analyzed using the Scion image analysis software (Scion, Frederick, MD). Total cell lysates were used to analyze pro-caspase-3, Bcl-2 and Bax expression and normalized with β-actin expression. Cytosolic and mitochondrial extracts were used to analyze the insection of Bax into mitochondria, and protein bands were compared with the expression of β-actin (marker of cytosolic fraction) and Complex I (marker of mitochondrial fraction).

### Statistical analysis

Values represent the mean ± s.e.m. of at least three independent experiments. Comparisons among groups were performed by using the Student's *t *test and One-way ANOVA using a SPSS 12.0 software.

## Competing interests

The author(s) declare that they have no competing interests.

## Authors' contributions

PNFL was responsible for designing, performing and writing the manuscript. DAR and SEM contributed to experiments of apoptosis by Hoechst and APOBrDU. DOC and GAR were responsible of experiments using Western blot analysis, and contributed to writing of the manuscript. MB, RL and MS performed and interpreted the flow cytometry experiments. LMP was responsible for the design and writing of the manuscript. All authors read and approved the final manuscript.
